# Network analysis of symptoms and psychological distress in patients with sudden sensorineural hearing loss

**DOI:** 10.1097/MD.0000000000050381

**Published:** 2026-08-28

**Authors:** Jiemei Wang, Yifang Li, Wei Song, Li Xie, Changju Tao

**Affiliations:** a candidate, School of Nursing, Anhui University of Chinese Medicine, Hefei, Anhui, China; b School of Nursing, Anhui University of Chinese Medicine, Hefei, Anhui, China; c Laboratory of Geriatric Nursing and Health, Anhui University of Chinese Medicine, Hefei, Anhui, China; d Information Center, The First Affiliated Hospital of Anhui University of Chinese Medicine, Hefei, Anhui, China.

**Keywords:** anxiety, depression, hospital anxiety and depression scale (HADS), network analysis, sudden sensorineural hearing loss (SSNHL), symptoms

## Abstract

Sudden sensorineural hearing loss (SSNHL) is characterized by rapid-onset, unexplained hearing impairment, frequently accompanied by symptoms such as tinnitus, dizziness, and nausea. Patients with SSNHL often experience psychological distress, including anxiety and depression, which are interconnected, offering insights for holistic management. This study aims to elucidate these connections, with a focus on anxiety and depression, using network analysis to inform holistic management strategies. Network analysis was employed to model symptom-psychological interactions, identifying central nodes and their connectivity patterns. Key metrics included node strength, centrality, and edge weights to quantify associations between symptoms and psychological subscales. Network analysis revealed significant correlations between SSNHL symptoms (e.g., tinnitus, dizziness) and psychological subscales Hospital Anxiety and Depression Scale ([Hospital Anxiety and Depression Scale)-anxiety, Hospital Anxiety and Depression Scale-depression]). Key nodes with high centrality included anxiety, depression, and dizziness, demonstrating significant interconnections. Anxiety exhibited the highest node strength, indicating its pivotal role in the symptom-psychological network. Edge weights highlighted bidirectional relationships, particularly between anxiety and tinnitus, as well as depression and nausea. This study underscores the critical role of anxiety and depression in the symptom network of SSNHL, emphasizing their association with disease progression and patient well-being. It is important to note that, due to the cross-sectional design, these findings represent an exploratory model of associations rather than causal relationships. The application of network analysis has given us a deeper understanding of the multifaceted nature of SSNHL, enabling us to develop more personalized and effective treatment strategies.

## 1. Introduction

Sudden sensorineural hearing loss (SSNHL), clinically defined as an abrupt hearing reduction of ≥30 dB across at least 3 consecutive frequencies within 72 hours, represents an otologic emergency with idiopathic etiology. This rapid-onset auditory impairment is frequently accompanied by vestibular symptoms including nausea, vomiting, and vertigo, as well as persistent tinnitus.^[1–6]^ The condition’s sudden manifestation not only disrupts auditory function but also imposes significant psychosocial burdens, compromising communication capacity, occupational performance, and overall quality of life.^[2]^ SSNHL is not only a physical ailment but can also have profound emotional consequences, including feelings of anxiety and depression due to the sudden and often unexplained nature of the hearing loss.^[7]^ Therefore, timely intervention is paramount in cases of SSNHL to optimize recovery outcomes and enhance the quality of life for affected individuals.

Notably, patients demonstrate heightened susceptibility to anxiety disorders and depression, with prevalence rates exceeding general population benchmarks with SSNHL.^[8]^ The diagnostic triad of sudden hearing loss, persistent tinnitus, and vestibular dysfunction creates a pathogenic milieu for psychological distress, characterized by acute fear responses, perceived helplessness, and anticipatory anxiety regarding prognosis.^[9]^ Furthermore, chronic auditory deprivation and social interaction impairments may potentiate depressive symptomology,^[10]^ and persistent symptoms like tinnitus and dizziness, can contribute to the development of depressive symptoms.^[11,12]^

Comprehensive psychological assessment constitutes an essential component of SSNHL management, with anxiety and depression screening serving as critical prognostic indicators.^[7,13,14]^ The Hospital Anxiety and Depression Scale (HADS), a validated psychometric instrument, has been extensively implemented for quantifying affective disorder severity in medically compromised populations.^[11,15,16]^ When applied in the context of SSNHL, the HADS can offer valuable insights into the psychological impact of this condition on affected individuals.^[11]^ Recent studies have emphasized the significance of utilizing the HADS in the evaluation and management of anxiety and depression in SSNHL patients. HADS provides a structured approach to addressing the psychological aspects of the condition, which consists of 2 subscales. By quantifying the psychological distress experienced by patients, the HADS scores provide valuable insights into the impact of emotional well-being on the overall treatment outcomes and quality of life in individuals with SSNHL.

Recently, researchers have proposed that network models can offer a comprehensive characterization of psychological syndromes.^[17]^ According to network theory, psychological disorders can be conceptualized as a complex system of interconnected symptoms that mutually amplify, reinforce, and sustain each other.^[18–20]^ Network analysis highlights clusters of strongly interconnected symptoms and numerical rating scales; meanwhile, it quantifies the relative importance of individual symptoms. Network analysis has been used in the medical field. Such as, it has been used to describe the symptoms and progression of schizophrenia,^[21]^ alcohol disorder,^[22]^ somatic symptom disorder and panic disorder,^[23]^ and depression, anxiety related disorder.^[24–26]^ To describe the association within the psychological state and SSNHL, many researchers have conducted some assessment of mental health in patients with SSNHL.^[27–30]^ However, none of these have characterized the network structure of the HADS and the symptoms of the patients with SSNHL. Network analysis could provide unique insights into symptom and HADS, which can provide some advice for doctors.

Incorporating network analysis into the study of symptoms and psychological distress in SSNHL represents an approach to unraveling the intricate interplay between physical manifestations and emotional well-being in this population. The primary objective of this study is to systematically map the dynamic relationships among SSNHL-related symptoms and psychological subscales (specifically anxiety and depression) using network modeling, thereby identifying pivotal nodes that are central to symptom clusters and psychological distress. By integrating the HADS with clinical symptoms, this research addresses their interconnectedness. The significance of this work lies in the fact that: network analysis provides a holistic framework to quantify symptom centrality, prioritize intervention targets, and elucidate the complex interconnections between psychological states and physical symptoms within an exploratory network model. Given the cross-sectional nature of this study, our findings are hypothesis-generating rather than causal, providing a foundation for future longitudinal and interventional research. Furthermore, the identification of key nodes such as anxiety-related “butterflies in the stomach” (A7) and depression-linked “loss of interest in appearance” (D5) offers actionable pathways for personalized care, enabling clinicians to target symptom networks at their most influential points. This approach not only enhances prognostic accuracy but also lays the foundation for developing evidence-based guidelines tailored to the biopsychosocial complexity of SSNHL, ultimately improving long-term outcomes and quality of life for affected individuals.

## 2. Materials and methods

### 2.1. Patient selection and data availability

**Figure 1.**
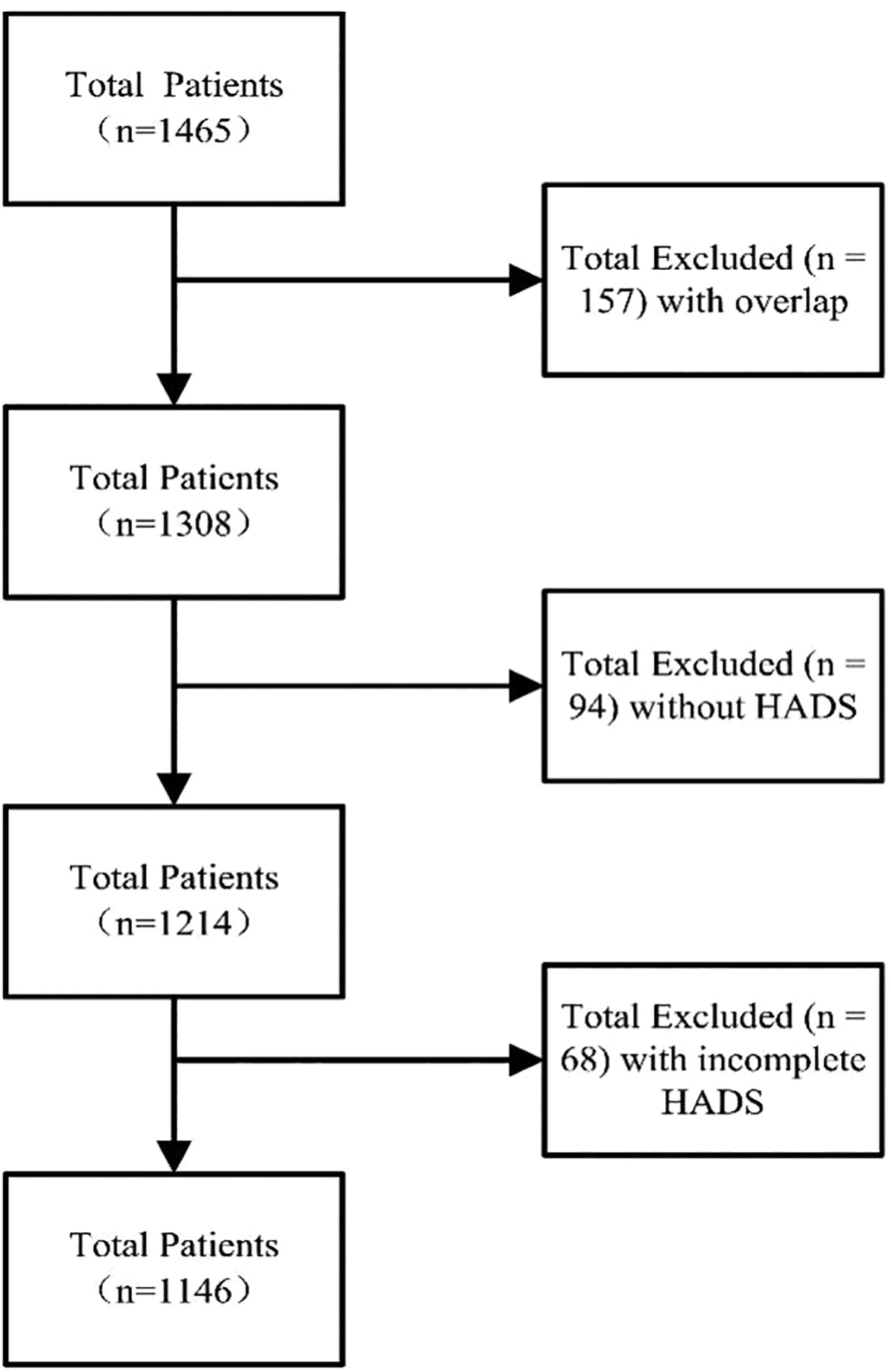
Patient selection diagram. HADS = Hospital Anxiety and Depression Scale.

Clinical data were retrospectively collected from patients treated at the Department of Otolaryngology, First Affiliated Hospital of Anhui University of Chinese Medicine, between January 2021 and December 2023. All participants were diagnosed with SSNHL according to the 2015 Chinese Guidelines for the Diagnosis and Treatment of SSNHL. During hospitalization, patients completed the HADS for self-assessment, while their medical histories and symptoms were evaluated by board-certified otolaryngologists. Inclusion criteria required: confirmation of SSNHL per the 2015 diagnostic guidelines; and absence of confounding conditions, including middle ear pathologies, acoustic neuroma, prior head trauma, ototoxic medication use, or significant noise exposure. Exclusion criteria comprised: transfer to another institution during treatment, concurrent diagnoses of acoustic neuroma or cerebral thrombosis, and incomplete or overlapping datasets (see Figure 1). This study received ethical approval from the Institutional Review Board of the First Affiliated Hospital of Anhui University of Chinese Medicine (2025AH-71).

### 2.2. Measures

#### 2.2.1. Basic information

In order to examine patients’ demography, a new variable was calculated that contains sex, age, and years. In common sense, sex was male or female.

#### 2.2.2. Symptoms

In this paper, 6 symptoms were collected for each patient by professional otolaryngologists through the SSNHL diagnosis and treatment guidelines (2015). These symptoms were “Hearing Loss,” “Tinnitus,” “Aural Fullness,” “Dizziness/Vertigo,” “Sleep Disturbance/ Insomnia,” and “Nausea/Vomiting.” Each symptom was categorized as either “Yes” or “No,” represented by 1 and 0, respectively.

### 2.3. Characterization variables (HADS)

As mentioned above, the HADS developed by British psychiatrists Zigmond and Snaith in 1983, is a validated self-report instrument widely utilized for screening anxiety and depressive symptoms in clinical populations. Comprising 2 distinct subscales, the Anxiety Subscale (HADS-A) and the Depression Subscale (HADS-D), the HADS includes 14 items (7 per subscale) rated on a 4-point Likert scale (0–3). Items assess symptom frequency over the preceding week, with certain questions reverse-scored to mitigate response bias. Subscale scores are calculated by summing individual item responses, yielding total scores ranging from 0 to 21 for anxiety (HADS-A) and depression (HADS-D), where higher scores reflect greater symptom severity.

A validated cutoff score of ≥9 on either subscale optimizes sensitivity and specificity for identifying clinically significant anxiety or depression, with scores ≤8 categorized as nonclinical. This threshold facilitates efficient triage of psychological distress in hospital settings, aligning with the tool’s original purpose as a screening aid rather than a diagnostic instrument.

### 2.4. Statistical analysis

After selection, the number that meets the inclusion criteria is 1146. The Table 1 shows demographic and descriptive data related to the symptoms. Meanwhile, the details of the HADS were described in Table 2.

**Table 1:** The demographic and descriptive data related to the symptoms.

Variables		Ration
Age(M[SD])	38.52 ± 14.99	
Sex		
Male	535	46.68%
Female	611	53.32%
Education yr (M[SD])	11.96 ± 4.12	
Symptoms		
Hearing Loss	1141	99.56%
Tinnitus	1078	94.07%
Aural fullness	124	10.82%
Dizziness/Vertigo	127	11.08%
Sleep disturbance/insomnia	55	4.8%
Nausea/vomiting	22	1.92%

M(SD) = Mean(Standard Deviation).

**Table 2:** The details of HADS.

HADS	Num (Ration)				Total score ≥9	Total score (M[SD])
Anxiety Subscale	0	1	2	3		
Feel tense or wound up	431	649	50	16	203 (17.71%)	4.73 ± 3.876
Get a sort of frightened feeling as if something awful is about to happen	489	442	197	18		
Worrying thoughts go through my mind	639	380	79	48		
Can sit at ease and feel relaxed	472	417	222	35		
Feel a trembling sense of fear	716	176	244	10		
Feel restless as if I have to be on the move	571	464	78	33		
Get a sort of frightened feeling, like “butterflies” in the stomach	606	426	92	22		
Depression subscale	0	1	2	3	Total score ≥9	Total score (M[SD])
Still enjoy the things I used to enjoy	523	429	133	61	166 (11.25%)	4.69 ± 3.619
Can laugh and see the funny side of things	641	259	190	56		
Feel cheerful	556	500	72	18		
Feel as if I have slowed down	411	633	89	13		
Have lost interest in my appearance	599	444	88	15		
Look forward with enjoyment to things	676	313	139	18		
I can enjoy a good book or radio or TV program	605	362	69	110		

HADS = Hospital Anxiety and Depression Scale, M(SD) = Mean (Standard Deviation).

### 2.5. Analyses

#### 2.5.1. Network estimation

Network estimation was performed using R software (version 4.3.3).^[31]^ The symptom and HADS subscale network structure was modeled via the Enhanced Least Absolute Shrinkage and Selection Operator algorithm,^[32]^ implemented with the qgraph (v1.9.8)^[33]^ and bootnet packages (Version 1.6).^[34]^ To optimize sparsity and model parsimony, the penalty parameter (γ=0.5) and the Extended Bayesian Information Criterion (EBIC) were applied, ensuring robust selection of node-specific neighbor relationships. Nodes (representing symptoms, HADS subscales, and demographic covariates) were interconnected via edges weighted by partial correlation coefficients, with edge thickness proportional to association strength and color denoting directionality (blue: positive, red: negative). Demographic covariates including age (B1), sex (B2), and education years (B3) were included as additional nodes in the network to statistically adjust for their potential confounding effects on the associations between symptoms and psychological subscales. The Fruchterman-Reingold algorithm positioned nodes with stronger and more frequent connections closer to one another, yielding a consolidated network topology.

Network analysis provides quantitative centrality indicators for each node based on the unique configuration of a network. In network analysis, centrality measures include Strength, Closeness, Betweenness, and Expected Influence. Strength represents the sum of the weights of all direct connections between a specific symptom and other nodes. Betweenness centrality quantifies the frequency with which a node (e.g., a symptom) acts as a bridge along the shortest paths connecting other node pairs. Closeness centrality, conversely, measures the proximity of a node to all others in the network, calculated as the reciprocal of the sum of its shortest path distances to every other node. These centrality metrics are conventionally standardized using z-scores to facilitate cross-network comparisons. Expected Influence, on the other hand, captures the anticipated impact or influence of a node on other nodes within the network, taking into account its connections, their strength, and its position within the network structure.

To address potential concerns about applying EBICglasso to binary symptom variables, we conducted a sensitivity analysis using the Ising model, implemented via the IsingFit package (IsingFit function). The Ising model is specifically designed for network estimation from binary data. Consistency between the 2 methods was assessed using Pearson and Spearman correlations of edge weights and node strength centrality.

#### 2.5.2. Robustness evaluation of symptom network metrics

The robustness of network attributes was evaluated through case-dropping bootstrap analyses. Network stability was operationalized as the resilience of centrality indices (e.g., betweenness, closeness) to systematic subsampling, where minimal deviation in node centrality under progressive data exclusion (e.g., up to 75% of cases) indicated high stability. This was quantified using the correlation stability coefficient, with values ≥0.5 considered acceptable. Edge weight accuracy was assessed via nonparametric bootstrapping (2500 iterations), generating 95% confidence intervals for each association. Bootstrap difference tests further compared edge weight magnitudes to identify statistically significant variations in network topology.

#### 2.5.3. Centrality metrics in triage patients

**Figure 2.**
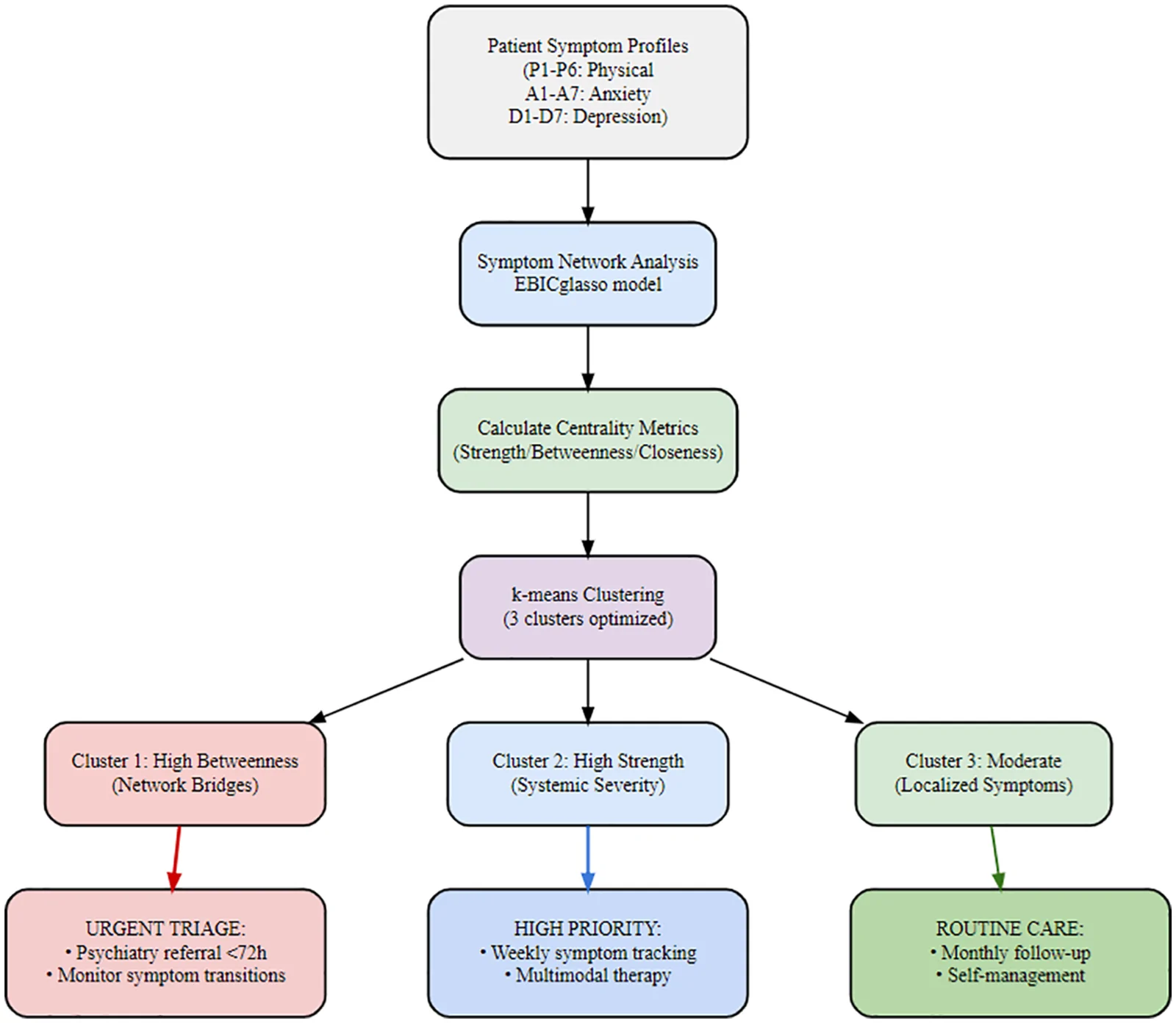
The workflow diagram of the classification process.

The three core indicators have been obtained through calculation, namely Strength, which represents the combined influence of symptom severity and network connectivity; Closeness, representing the efficiency of information transmission between symptoms; and Betweenness, which signifies the bridging effect connecting different symptom groups. The K-means algorithm is used to classify patients into 3 categories based on centrality metrics: High, Routine, and Urgent. The classification logic involves identifying clusters with the highest betweenness centrality (bridge-type patients), clusters with the highest strength centrality (patients with severe symptom burden), and the remaining patients categorized as Moderate. The workflow diagram of the classification process is shown in Figure 2.

## 3. Results

As shown in Table 1, 6 symptoms were collected. The main symptoms were “Hearing Loss” and “Tinnitus,” accounting for 99.56% and 94.07%, respectively. In additional, the ration of the total score >9 was 17.71% and 11.25%. See Tables 1 and 2 for additional sample characterization.

### 3.1. Network structure and centrality measure analysis

**Figure 3.**
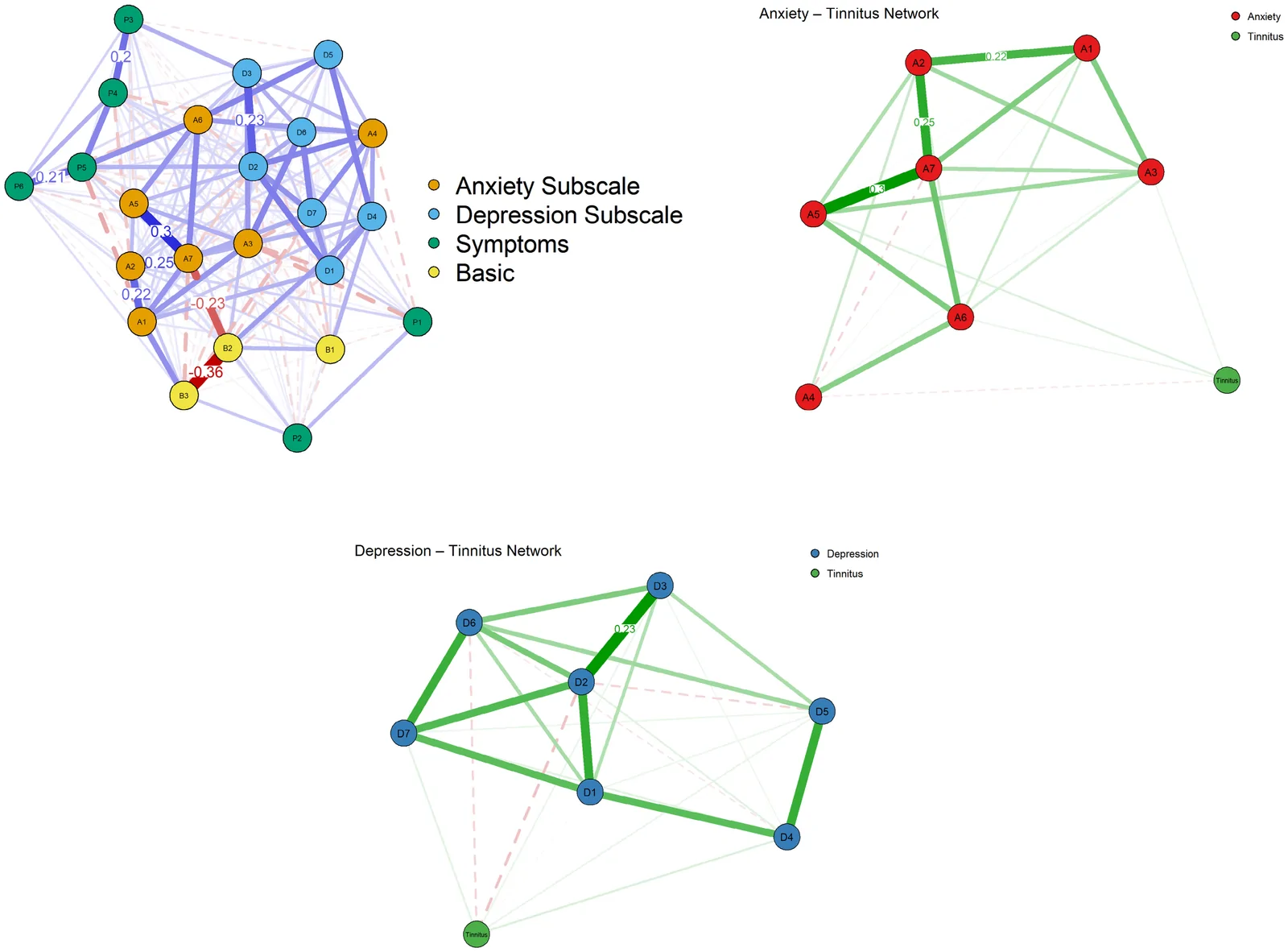
(A) Network structure of HADS items, SSNHL symptoms, and demographic covariates. The layout was generated using the Fruchterman-Reingold algorithm, which positions nodes with stronger and more frequent connections closer together. Colors denote node categories: yellow for HADS-Anxiety items (A1–A7), blue for HADS-Depression items (D1–D7), green for SSNHL symptoms (P1–P6), and gray for demographic covariates (B1 = age, B2 = sex, B3 = education years). Demographic covariates were included as additional nodes in the network estimation to adjust for their potential confounding effects on symptom–psychological associations; their placement in the periphery of the network reflects their relatively weaker connections to the core symptom–psychological structure. Edges represent partial correlation coefficients, with blue edges indicating positive associations and red edges indicating negative associations. Edge thickness is proportional to the strength of the association. (B) Network structure of anxiety-tinnitus. (C) Network structure of depression-tinnitus.

**Figure 4.**
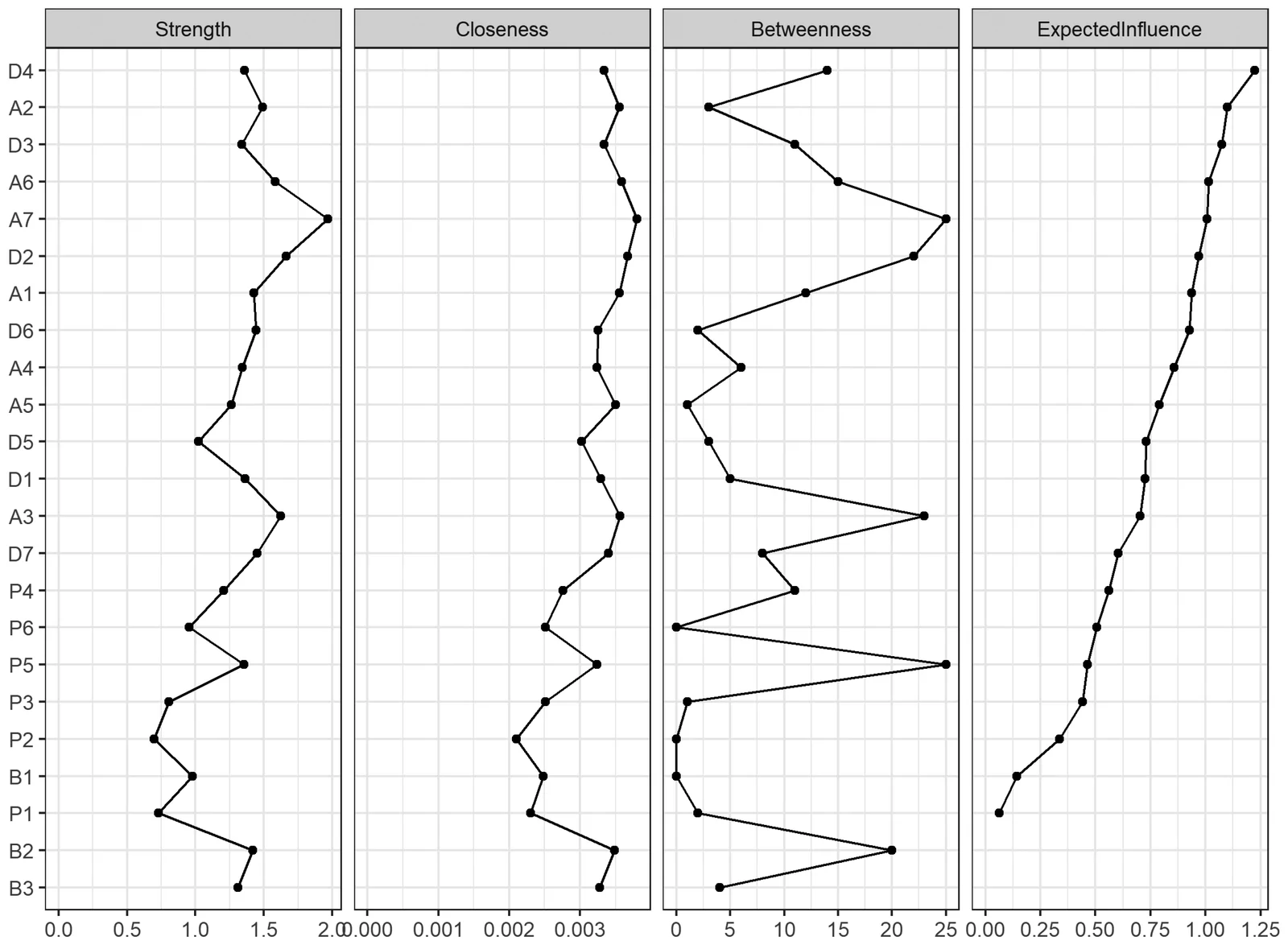
Centrality measures of all symptoms within the network.

**Figure 5.**
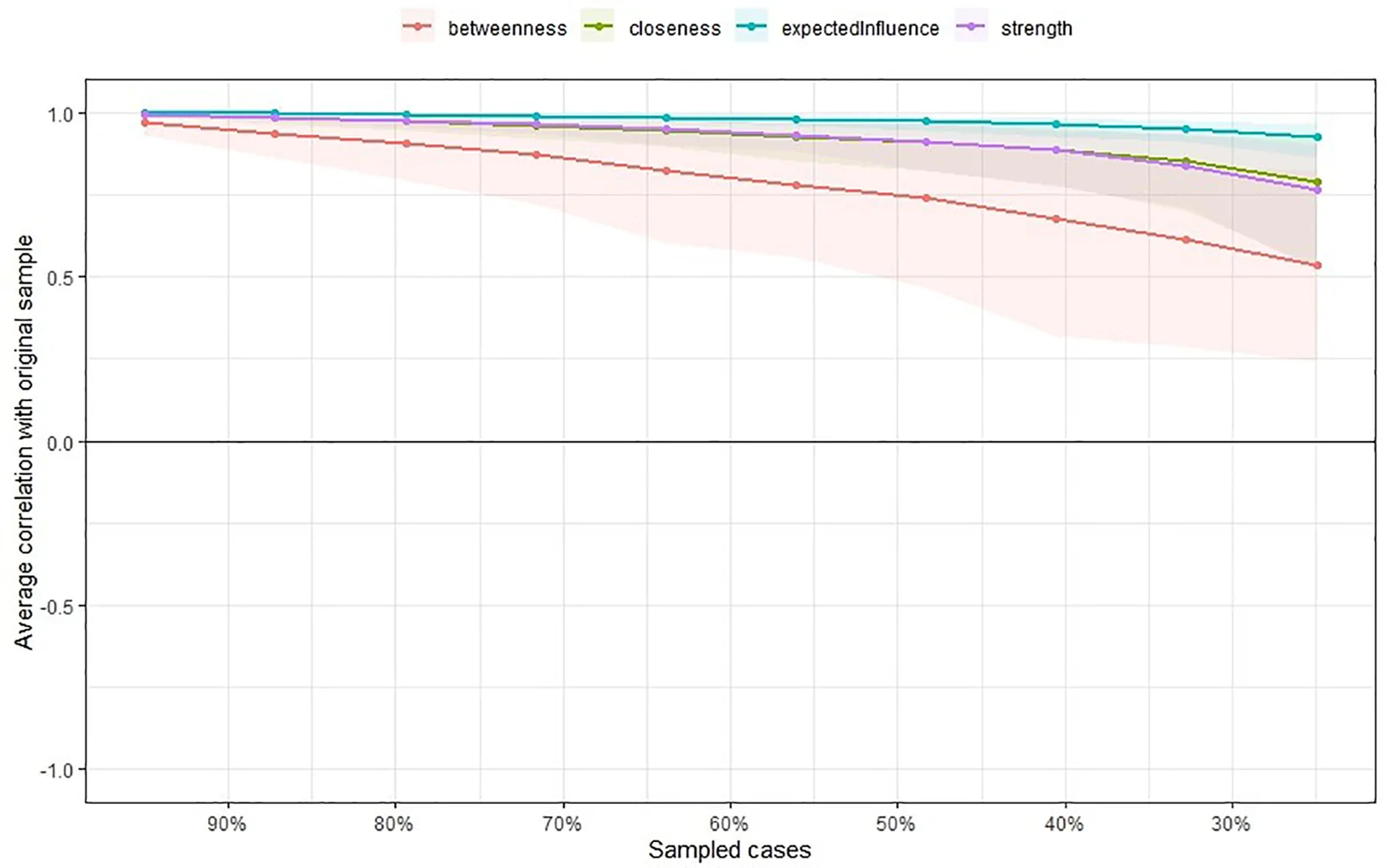
Stability of centrality indices by case dropping subset bootstrap.

The network was estimated with an EBICglasso model; the structure was shown in Figure 3A. 253 edges were retained (Mweight=0.029), and the subgroup analyses of anxiety-tinnitus and depression-tinnitus were shown in Figure 3–C. Figure 4 shows the network centrality index of Strength, Closeness, Betweenness, and Expected Influence. The stability of the network analysis was investigated, and it was found to have an excellent level of stability (i.e., CS=0.75). Figure 5 shows that 75% of the sample could be dropped without the network structure changing to a significant extent compared to the original structure.

With the Anxiety-subscale model, the node A5 (feel a trembling sense of fear) had the strongest connections with A7 (get a sort of frightened feeling like “butterflies” in the stomach) and A6 (feel restless as if I have to be on the move). Node P3(Aural Fullness) was connected with P4(Dizziness/Vertigo), P5(Sleep Disturbance/ Insomnia) and P6(Nausea/Vomiting). Node D3(feel cheerful) was connected with D2(can laugh and see the funny side of things), D5(have lost interest in my appearance) and A6(feel restless as if I have to be on the move). Nodes D7(I can enjoy a good book or radio or TV program), D2(can laugh and see the funny side of things), D4(feel as if I have slowed down) and A6(feel restless as if I have to be on the move) were connected with A4(can sit at ease and feel relaxed). Although A4 was situated at the periphery of the network, those nodes were directly connected.

In term of the strength, node A7 was most influential, as shown in Figure 4. Followed by A7, nodes D2, A3, and A6, all of which were also central characters of the patients with SSNHL. In contrast, some other symptoms were marginal, such as P1, P2, P3.

### 3.2. Network accuracy and stability

The network model demonstrated robust reliability, as evidenced by bootstrapped 95% confidence intervals for edge weights, with 2500 resampling iterations ensuring stability across parameter estimates, as shown in Figure 5. Bootstrap-based differential testing further confirmed statistically significant distinctions (α < 0.05) in the majority of edge weight comparisons, reinforcing the precision of inter-node associations.

### 3.3. Visualization of centrality metrics for triage patients

**Figure 6.**
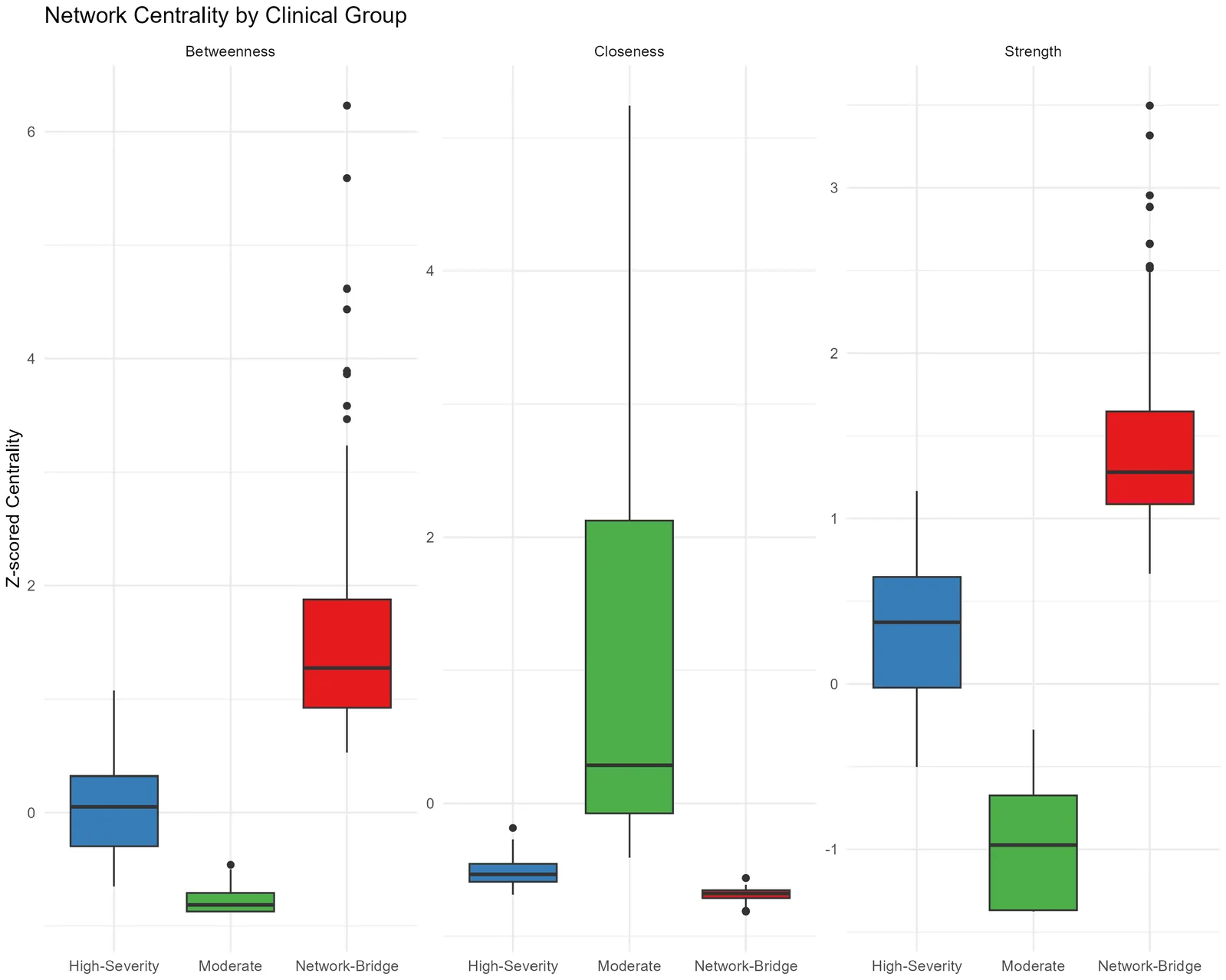
Network Centrality by clinic groups

**Figure 7.**
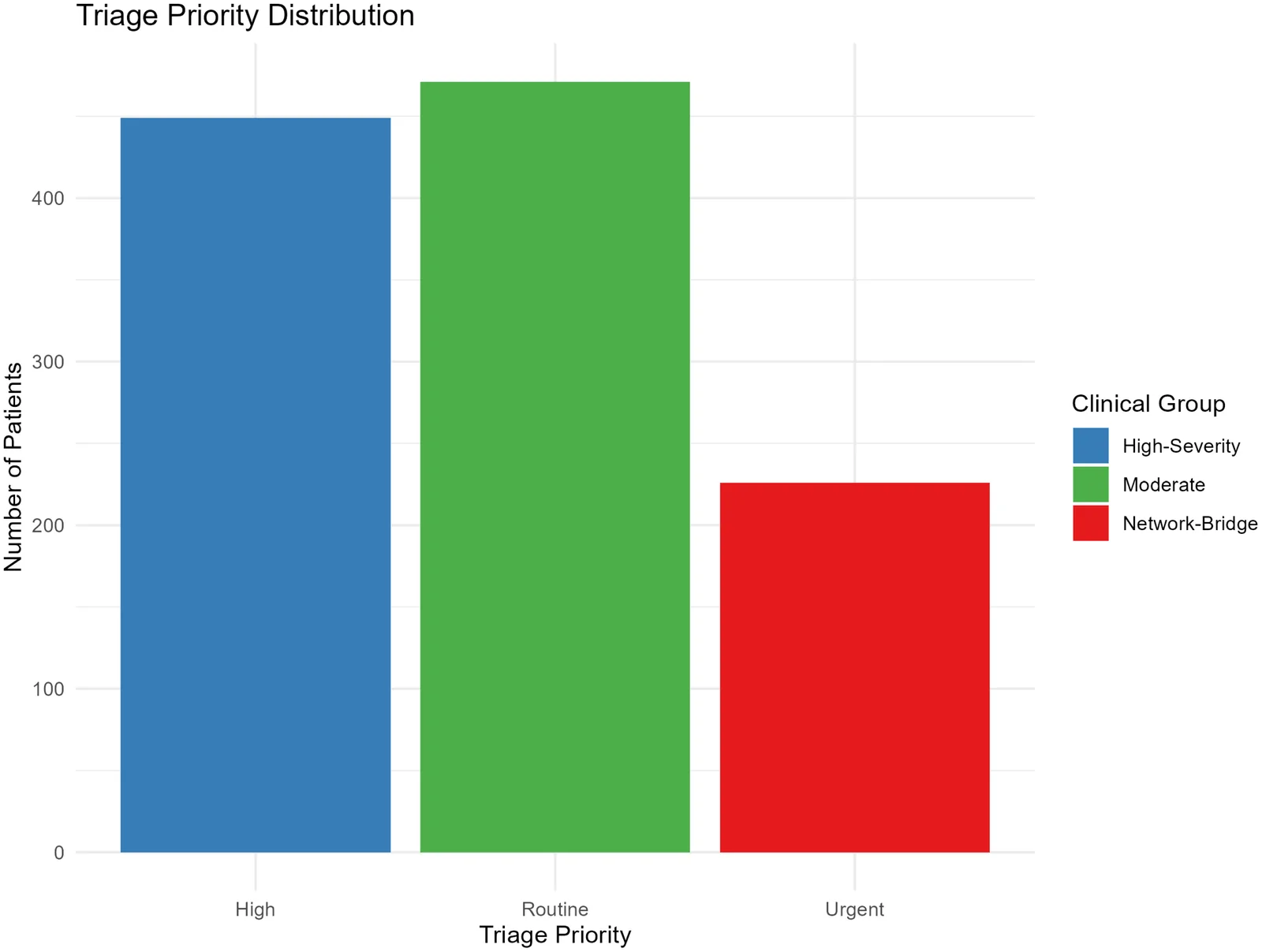
Distribution of Triage Priority

Visualization techniques were used to illuminate patient stratification patterns derived from network analysis. Figure 6 utilizes box plots to systematically compare distributions of 3 centrality metrics strength, betweenness, and closeness across the identified clinical categories. These plots reveal fundamental differences in network properties between subgroups: High-Bridge patients demonstrate significantly elevated betweenness centrality, while High-Severity cases show pronounced strength centrality. Complementarily, Figure 7 employs parallel bar charts to quantify the epidemiological distribution of the cohort across stratification categories. Color-coded bars (urgent→red, high→blue, routine→green) provide immediate clinical interpretation of symptom burden distribution.

### 3.4. Sensitivity analysis

The Ising model sensitivity analysis demonstrated high consistency with our primary EBICglasso estimates. Edge weights from the 2 methods showed strong correlation (Pearson *R* = 0.966, Spearman *R* = 0.864), and node strength centrality was nearly identical across methods (Pearson *R* = 0.987), supporting the robustness of our primary findings despite the binary nature of symptom variables.

## 4. Discussion

The present study employed network analysis to explore the interconnections between symptoms and psychological states, specifically anxiety and depression, in patients with SSNHL. By leveraging the HADS and clinical data from patients admitted to the Department of Otolaryngology at the First Affiliated Hospital of Anhui University of Chinese Medicine, we constructed a network that illustrates the complex interplay of symptoms associated with SSNHL. Our findings reveal a network structure characterized by significant associations among various symptoms and psychological subscales, offering novel insights into the condition.

The network demonstrated robust stability, with a correlation stability coefficient of 0.75 (Fig. 4), indicating that 75% of the sample could be excluded without significantly altering the network structure. This aligns with prior studies utilizing network analysis in psychiatric disorders, where CS ≥ 0.5 is considered acceptable for clinical interpretation.^[18–24]^ Centrality analysis (Fig. 3) identified anxiety-related node A7 (“butterflies in the stomach”) as the most influential, followed by D2 (“can laugh and see the funny side of things”) and A3 (“worrying thoughts”). These findings resonate with research by Zhao et al (2025), who similarly identified somatic anxiety symptoms (e.g., gastrointestinal distress) as central nodes in depression-anxiety networks among cardiovascular patients.^[24]^ The prominence of A7 underscores the physiological manifestations of anxiety in SSNHL, which may co-occur with subjective distress and impede recovery.

While hearing loss (P1, 99.56%) and tinnitus (P2, 94.07%) were nearly universal, less frequent symptoms such as dizziness/vertigo (P4, 11.08%) and nausea/vomiting (P6, 1.92%) formed distinct clusters with psychological subscales (Fig. 2). For example, dizziness/vertigo (P4) was strongly connected to aural fullness (P3) and sleep disturbance (P5), mirroring findings by Kim et al. (2020), who linked vestibular dysfunction to heightened anxiety in SSNHL cohorts.^[9]^ Notably, depressive symptoms (e.g., D5: “loss of interest in appearance”) showed direct connections to anxiety-related restlessness (A6), suggesting a bidirectional associations between emotional and physical symptoms. This aligns with Tomaz et al (2024), who reported that tinnitus severity mediated depressive symptoms in SSNHL patients.^[11]^

It is crucial to emphasize that, due to the cross-sectional design, these findings represent an exploratory network model of associations rather than a causal model. The centrality of a node does not imply it is a “cause” but rather a potentially important and influential hub within the symptom network that could be a promising target for intervention. Therefore, the identified central nodes, particularly A7 (“butterflies in the stomach”) and D2 (“can laugh and see the funny side of things”), should be viewed as priority targets for hypothesis-driven intervention studies, rather than as established causal drivers of the symptom network.

The centrality of A7 and D2 highlights actionable targets for intervention. For instance, cognitive-behavioral strategies targeting somatic anxiety (e.g., mindfulness for gastrointestinal distress) may be particularly relevant for addressing the A7-A5 (“trembling fear”) feedback loop. Similarly, enhancing social engagement (via D2 and D3: “feel cheerful”) may be relevant to addressing depressive symptoms linked to auditory deprivation, as proposed by Yuan et al. (2021) in qualitative studies of SSNHL coping mechanisms.^[27]^ These insights extend beyond traditional symptom-focused approaches, aligning with Goodwin et al (2023), who emphasized network-informed interventions in neurodegenerative disorders.^[20]^

An operational threshold of |Δ| > 0.5 was established, based on Tranchevent L et al.^[35]^ and validated through expert consultation. A difference of 0.5 standard deviations was determined to represent clinically significant separation in symptom impact. This study observed a node intensity difference between anxiety (mean = 0.599, SD = 0.227) and depression (mean = 0.775, SD = 0.281), yielding Δ = -0.176 (permutation test *P* = .239). The absolute difference (0.176) did not meet the predefined threshold of 0.5. These findings suggest that integrated therapy may be more appropriate than prioritizing interventions for a single symptom cluster (Beard et al 2016^[36]^).

The application of network analysis in this context represents a significant advancement in understanding the multifaceted nature of SSNHL. By elucidating the intricate web of symptoms and psychological states, healthcare professionals can design more personalized and effective treatment strategies that address both the physical and emotional aspects of the condition. Future research should continue to refine and expand upon these findings, incorporating larger sample sizes and potentially exploring dynamic changes in the network structure over time. Additionally, interventions tailored to the central nodes identified in our network could be evaluated for their efficacy in improving patient outcomes and quality of life.

In addition, the regression residual method was used to adjust all centrality metrics using, controlling for gender, age, and years of education. Key findings from this analysis include: demographic variables explained minimal variance; these covariates accounted for only 2% of symptom variation (average R^2^ = 0.02), suggesting their limited influence on network topology. Network stability after adjustment: A strong Pearson correlation (*R* = 0.64, *P* < .001) between unadjusted and adjusted networks confirms the robustness of core symptom associations. All of these indicate that the unadjusted network largely captures authentic symptom relationships, as covariate adjustments primarily refined demographically specific connections rather than altering fundamental patterns. Centrality indices demonstrate satisfactory robustness, with residual-based adjustments enhancing precision without disrupting core network architecture.

## 5. Limitations and future directions

While our large sample size (N = 1146) strengthens generalizability, several limitations warrant consideration. First, the cross-sectional design precludes causal inference; therefore, our findings are hypothesis-generating and should be tested in future longitudinal or interventional studies that can assess the temporal dynamics and causal relationships between these nodes.^[22]^ Second, the binary coding of symptoms (present/absent) may have obscured clinically meaningful severity gradations. Although this 1-hot encoding approach is the standard practice for symptom documentation in retrospective clinical research, and our Ising model sensitivity analysis confirmed the robustness of the network structure to this coding scheme, we acknowledge that this may indeed attenuate the true strength of associations between symptom severity and psychological states. For instance, the degree of hearing loss or the severity of tinnitus may have stronger or qualitatively different associations with anxiety/depression than their mere presence. Future studies should employ continuous measures, such as pure-tone averages for hearing loss, the Tinnitus Handicap Inventory for tinnitus severity, and the Dizziness Handicap Inventory for vertigo impact, to construct more nuanced network models that capture severity gradient information. Third, we did not collect data on corticosteroid treatment regimens (e.g., type, route, dosage, or timing), which represent a significant potential unmeasured confounder. Given the established role of corticosteroids in SSNHL management, treatment parameters could modulate the connections between physical symptoms and psychological distress. Prospective studies should systematically document treatment protocols, along with other potential confounders like audiometric data and comorbidities, to allow for a more robust and adjusted network analysis. Fourth, while our Ising model sensitivity analysis confirmed the robustness of EBICglasso estimates (*R* = 0.966), methods designed for mixed measurement levels may be optimal for future studies. Fifth, incorporating objective biomarkers (e.g., cortisol levels) and audiometric data (e.g., pure-tone average) would further strengthen causal inferences.^[1]^

## 6. Conclusions

In conclusion, our study demonstrates the feasibility and value of network analysis in studying the symptoms and psychological distress associated with SSNHL. It is crucial to emphasize that, due to the cross-sectional design, these findings represent an exploratory network model of associations rather than a causal model. The centrality of a node does not imply it is a “cause” but rather a potentially important and influential hub within the symptom network that could be a promising target for intervention. The insights gained from this analysis contribute to a deeper understanding of the condition and hold promise for guiding more effective and holistic care for individuals affected by SSNHL.

## Acknowledgments

The authors extend their appreciation to the Laboratory of Geriatric Nursing and Health, Anhui University of Chinese Medicine, for funding this work.

## Author contributions

**Software:** Jiemei Wang, Wei Song.

**Conceptualization:** Yifang Li, Li xie.

**Funding acquisition:** Yifang Li.

**Methodology:** Yifang Li.

**Investigation:** Changju Tao.
